# Dataset on structure, stability and myocardial effects of a new hybrid aspirin containing nitrogen monoxide-releasing molsidomine moiety

**DOI:** 10.1016/j.dib.2019.104146

**Published:** 2019-06-12

**Authors:** Kitti Szőke, Attila Czompa, István Lekli, Péter Szabados-Fürjesi, Mihály Herczeg, Magdolna Csávás, Anikó Borbás, Pál Herczegh, Árpád Tósaki

**Affiliations:** a Department of Pharmacology, Faculty of Pharmacy, University of Debrecen, Debrecen, Hungary; b Department of Bioanalytical Chemistry, Faculty of Pharmacy, University of Debrecen, Debrecen, Hungary; c Department of Pharmaceutical Chemistry, Faculty of Pharmacy, University of Debrecen, Debrecen, Hungary

**Keywords:** ERJ-500, Hybrid aspirin, Molsidomine

## Abstract

Herein ^1^H and ^13^C NMR spectra of ERJ-500, a new hybrid aspirin derivative, covalently conjugated to nitrogen monoxide donor linsidomine are presented as well as NMR spectra of its synthetic intermediate compounds. HPLC-MS measurements data are also included, demonstrating the stability of the linsidomine-aspirin hybrid in oxidation reactions. This data article also concerns miscellaneous myocardial parameters of isolated rat hearts as a complementation of the tables shown in the paper entitled “A new, vasoactive hybrid aspirin containing nitrogen monoxide-releasing molsidomine moiety” Szoke et al., 2019. Column tables represent data of aorta flow, aortic pressure, derivated aortic pressure and cardiac output.

**Table:** Specifications table

Subject area	*Chemistry, Biology*
More specific subject area	*Organic chemistry, Pharmacology*
Type of data	^*1*^*H and*^*13*^*C NMR spectra, HPLC-MS chromatograms, column tables*
How data was acquired	*NMR Bruker DRX-400 spectrometer at 25 °C, HPLC-MS*LTQ XL linear ion trap mass spectrometer coupled with Accela LC system (Thermo Fisher Scientific, Waltham, MA, USA).*, “Isolated working heart system”.*
Data format	*Raw, filtered and analyzed*
Experimental factors	*Initial compounds were purchased, the intermediates and the end-product were synthesized as described in the original paper.*
Experimental features	*New compounds have been characterized by spectrometric methods and an ex vivo technique on rat hearts*
Data source location	- Department of Pharmacology and Department of Bioanalytical Chemistry, Nagyerdei Krt 98, H-4032 Debrecen, Hungary - Department of Pharmaceutical Chemistry, Faculty of Pharmacy, University of Debrecen, Egyetem tér 1, H-4032 Debrecen, Hungary
Data accessibility	*Data are provided with this article.*
Related research article	K. Szoke, A. Czompa, I. Lekli, P. Szabados-Furjesi, M. Herczeg, M. Csavas, A. Borbas, P. Herczegh, A. Tosaki, A new, vasoactive hybrid aspirin containing nitrogen monoxide-releasing molsidomine moiety, Eur. J. Pharm. Sci., 131, 2019, 159–166 [1].

**Table:** 

**Value of the data** • The NMR spectra can be used for structure elucidation of similar synthetic compounds. • Dataset of stability tests could be used to pretest the degradation profiles of molecules planned to try in vivo circumstances. • The cardiac functions detailed here are important indicator related to the contractile activity of the myocardium (see Fig. 2).

## 1 Data

**Fig. 1:**
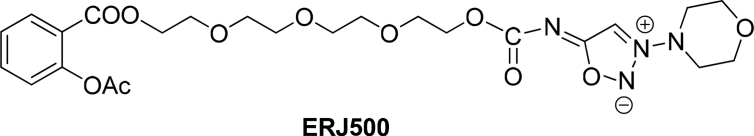
Structure of ERJ-500.

**Fig. 2:**
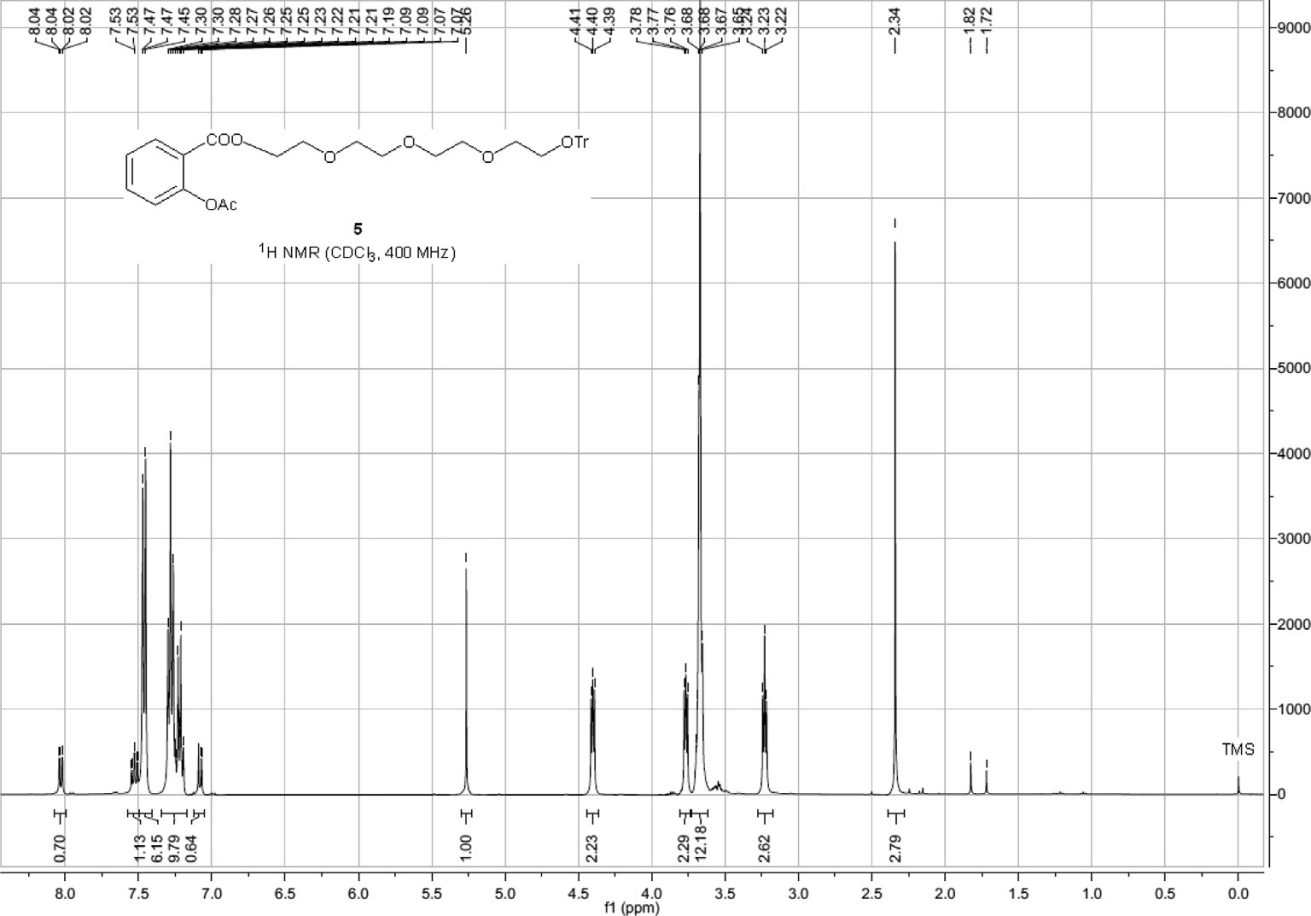
^1^H NMR spectra of compound **5**.

Spectra from ^1^H and ^13^C NMR measurements are reported to prove the structure of the synthesized compounds [1] (see Fig. 1, Fig. 2, Fig. 4, Fig. 5, Fig. 6, Fig. 7, Fig. 8, Fig. 9). HPLC-MS measurements were used to study the oxidative stability of the compound ERJ-500, representative total ion chromatogram of the oxidation by synthetic porphyrin and the Chemical Fenton System can be seen (Fig. 10, Fig. 11). Cardiac parameters (*aorta flow, aortic pressure, derivated aortic pressure, and cardiac output*) were registered (Fig. 12) in “isolated working hearts” treated with **ERJ-500**.

**Fig. 3:**
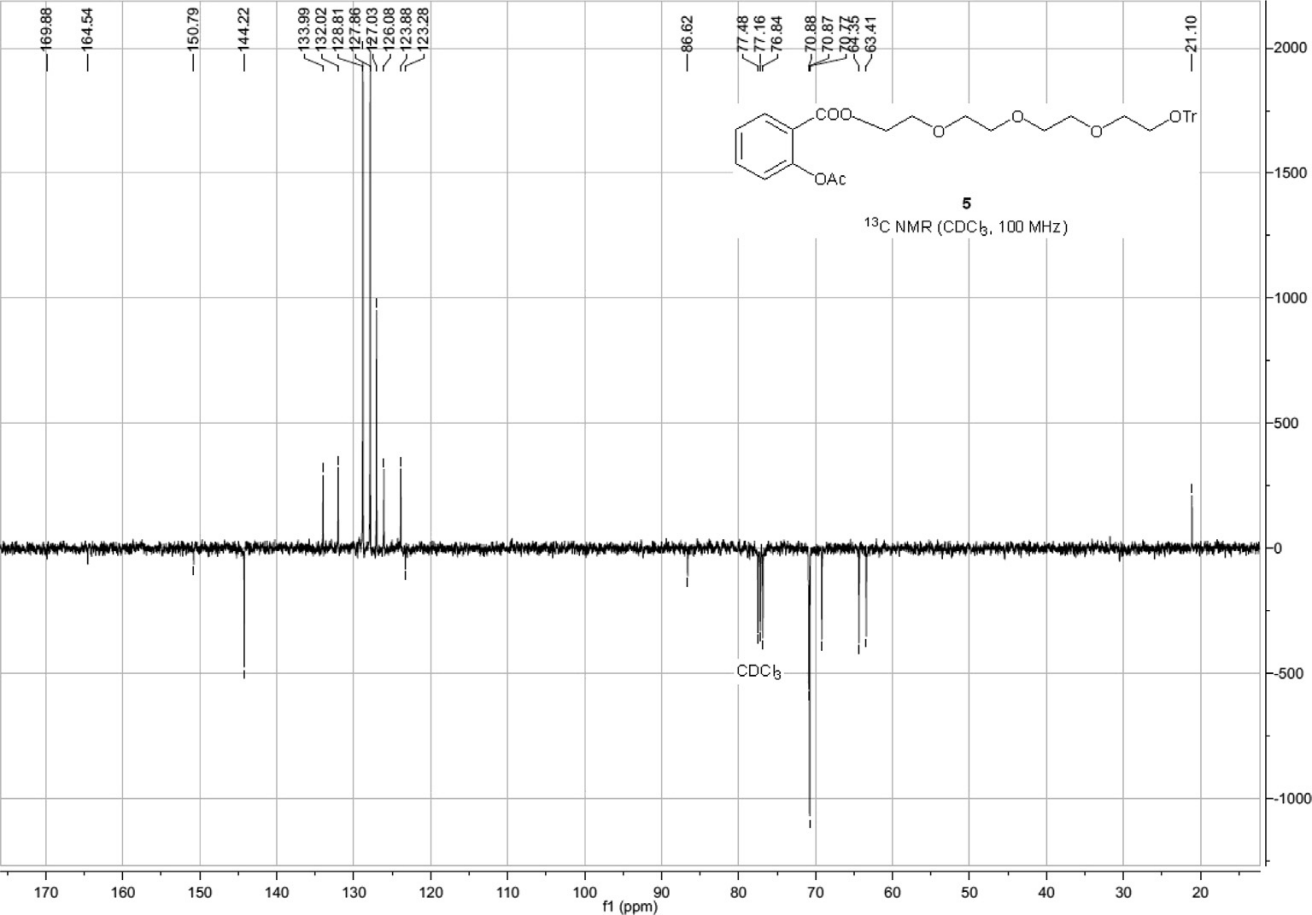
^13^C NMR spectra of compound **5**.

**Fig. 4:**
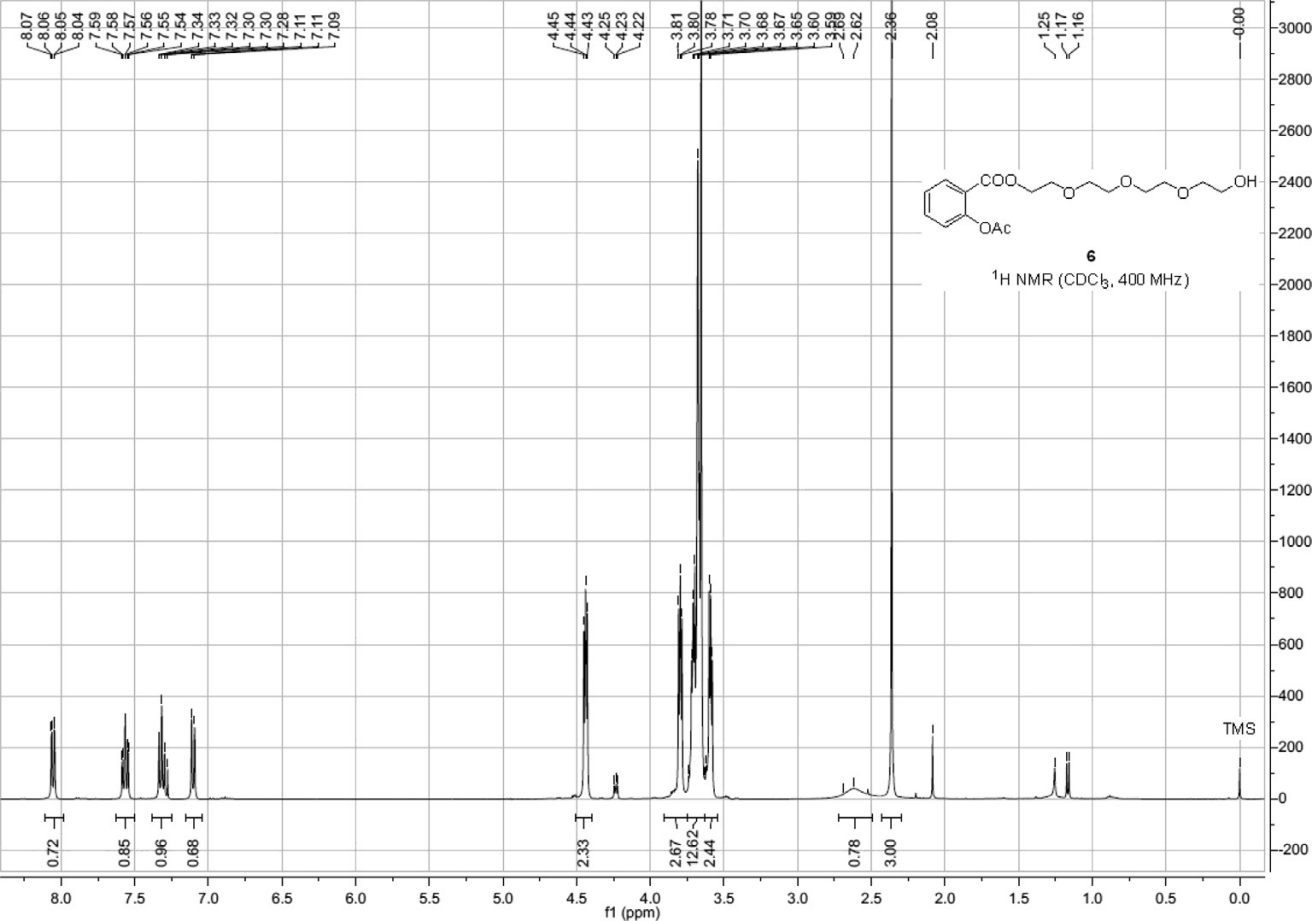
^1^H NMR spectra of compound **6**.

**Fig. 5:**
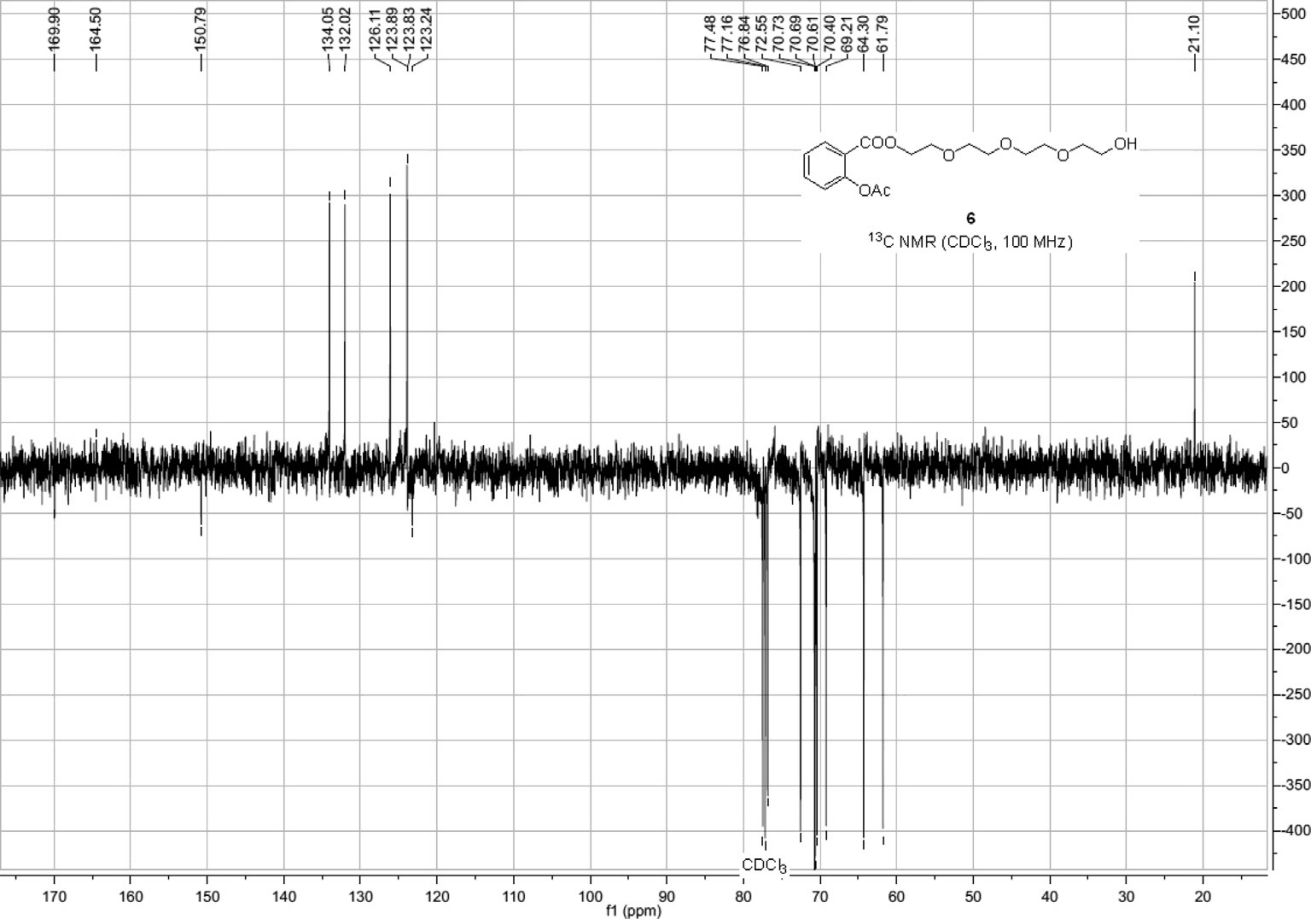
^13^C NMR spectra of compound **6**.

**Fig. 6:**
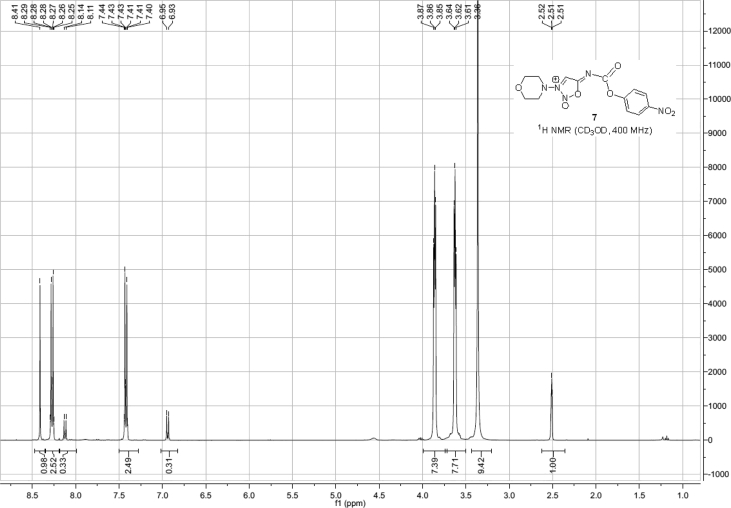
^1^H NMR spectra of compound **7**.

**Fig. 7:**
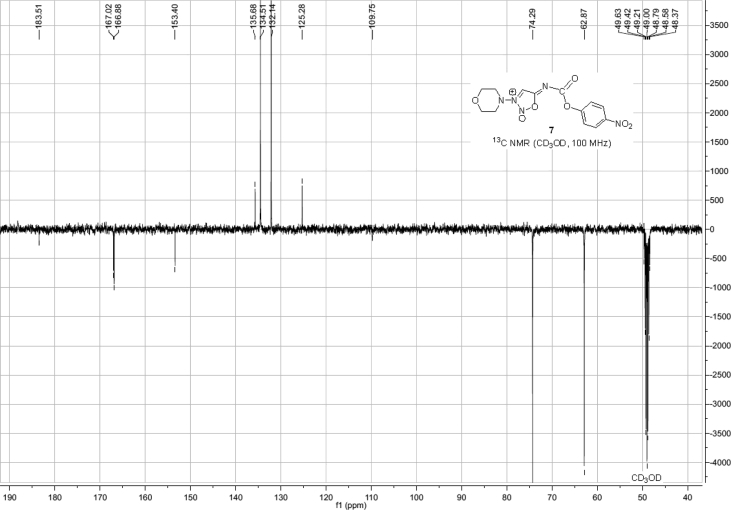
^13^C NMR spectra of compound **7**.

**Fig. 8:**
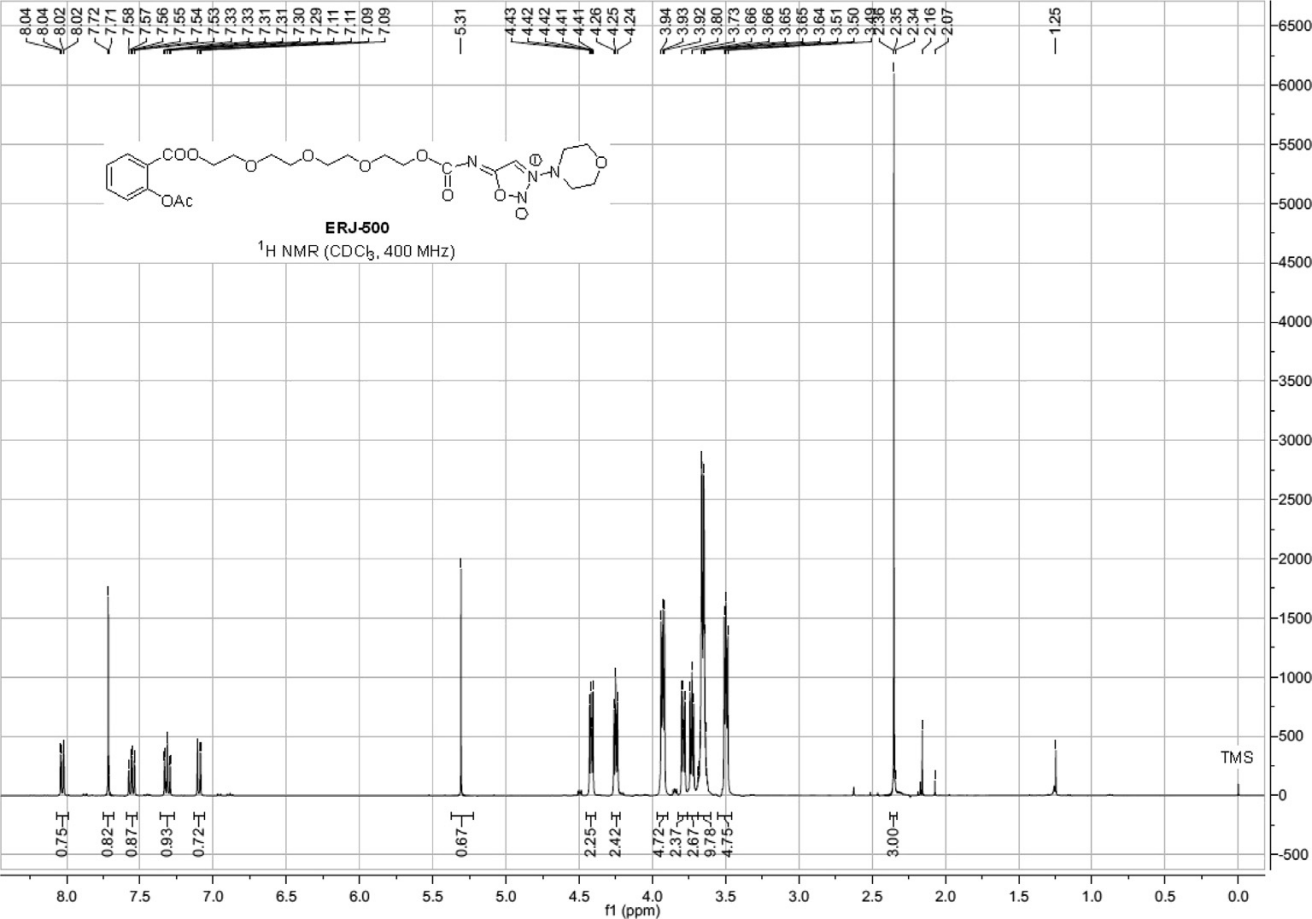
^1^H NMR spectra of compound **ERJ-500**.

**Fig. 9:**
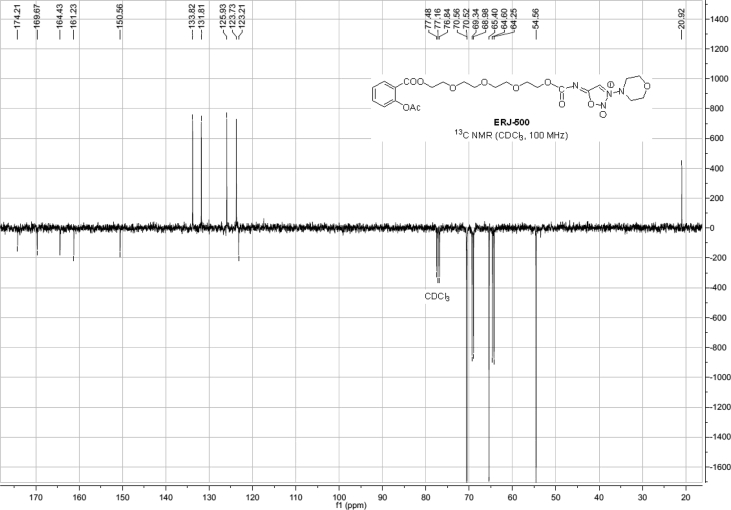
^13^C NMR spectra of compound **ERJ-500**.

**Fig. 10:**
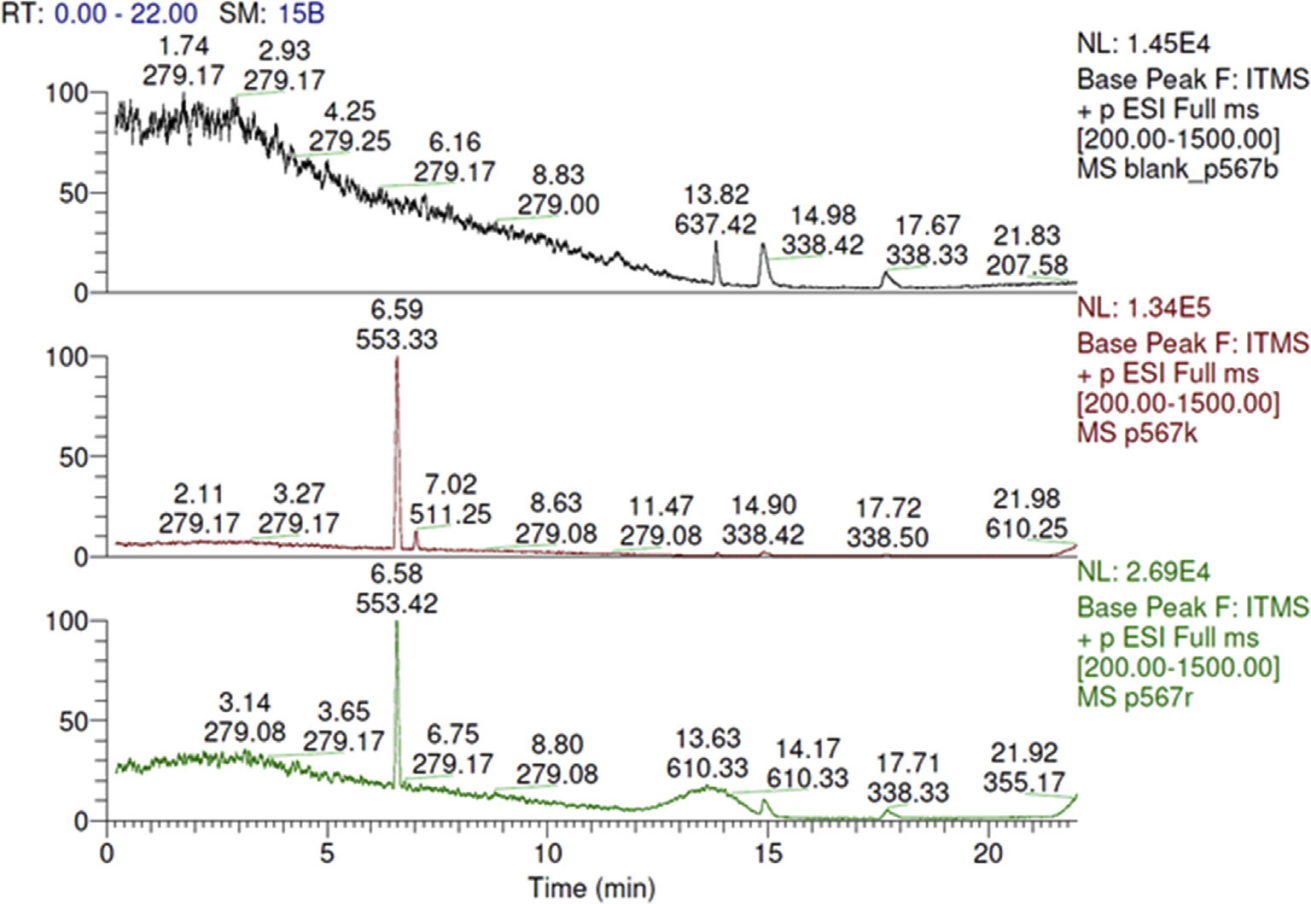
Representative total ion chromatogram of the oxidation by synthetic porphyrin. On the control chromatogram (red) the peak at 6.59 represents **ERJ-500**. After oxidation (green chromatogram) the peak of **ERJ-500** at 6.58 remained unchanged.

**Fig. 11:**
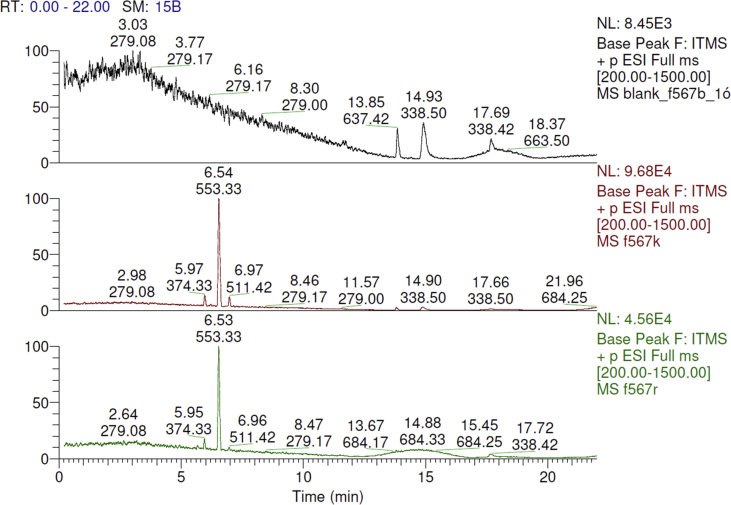
Representative total ion chromatogram of the oxidation by the Chemical Fenton System. On the control chromatogram (red) the peak at 6.54 represents **ERJ-500**. After oxidation (green chromatogram) the peak of **ERJ-500** at 6.53 remained unchanged.

**Fig. 12:**
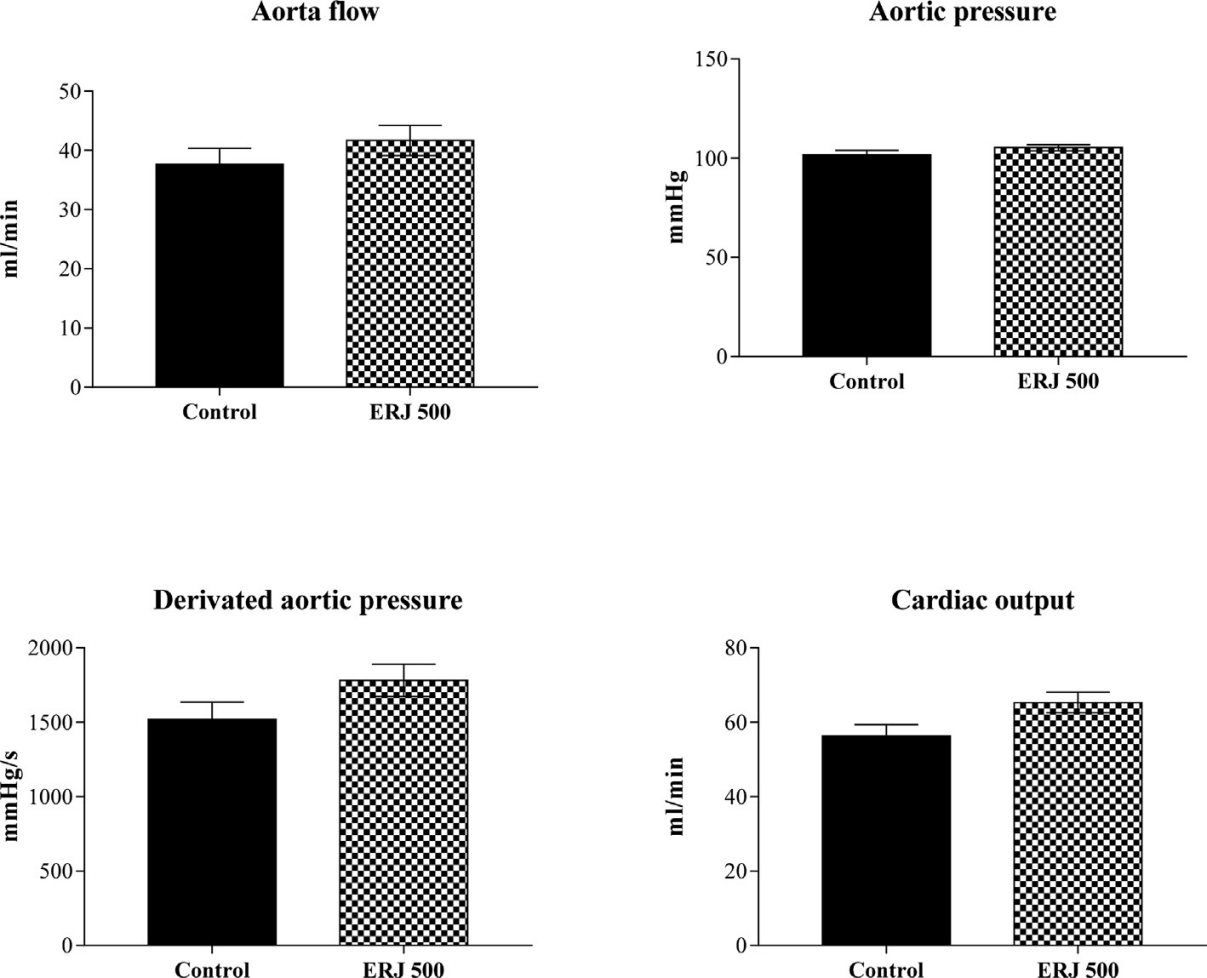
Myocardial function. The results show aorta flow, aortic pressure, derivated aortic pressure, and cardiac output in control and ERJ-500 treated hearts.

### 1.1 Characterization of new compounds

The ^1^H NMR (400 MHz) and ^13^C NMR (101 MHz) spectra were recorded with a Bruker DRX-400 spectrometer at 25 °C. Chemical shifts are referenced to Me_4_Si (0.00 ppm for ^1^H) and to the residual solvent signals (CDCl_3_: 77.1 for ^13^C).

### 1.2 Compound 5

^1^H NMR (400 MHz, CDCl_3_): *δ* 8.03 (dd, *J* = 7.8 Hz, *J* = 1.8 Hz, 1H, arom), 7.53 (td, *J* = 7.8 Hz, *J* = 1.8 Hz, 1H, arom), 7.47–7.45 (m, 6H, arom), 7.30–7.19 (m, 10H, arom), 7.08 (dd, *J* = 8.1 Hz, *J* = 0.8 Hz, 1H), 4.41–4.39 (m, 2H, TEG-*C*H_2_), 3.78–3.76 (m, 2H, TEG-*C*H_2_), 3.70–3.65 (m, 10H, 5 × TEG-*C*H_2_), 3.23 (t, *J* = 5.2 Hz, 2H, TEG-*C*H_2_), 2.34 (s, 3H, *C*H_3_ Ac); ^13^C NMR (101 MHz, CDCl_3_): *δ* 169.9 (1C, C_q_ Ac), 164.5 (1C, *C*OO), 150.8 (1C, C_q_ arom), 144.2 (3C, C_q_ arom), 134.0, 132.0, 128.8, 127.9, 127.0, 126.1, 123.9 (19C, arom), 123.3 (1C, Cq arom), 86.6 (1C, C_q_ Tr), 70.9, 70.8, 70.7, 69.2, 64.4, 63.4 (8C, 8 × TEG-*C*H_2_), 21.1 (1C, *C*H_3_ Ac).

### 1.3 Compound 6

^1^H NMR (400 MHz, CDCl_3_): *δ* 8.05 (dd, *J* = 7.8 Hz, *J* = 1.6 Hz, 1H, arom), 7.56 (td, *J* = 7.9 Hz, *J* = 1.6 Hz, 1H, arom), 7.32 (td, *J* = 7.6 Hz, *J* = 1.2 Hz, 1H, arom), 7.11 (dd, *J* = 8.1 Hz, *J* = 1.2 Hz, 1H, arom), 4.45–4.43 (m, 2H, TEG-*C*H_2_), 3.81–3.78 (m, 2H, TEG-*C*H_2_), 3.74–3.65 (m, 10H, 5 × TEG-*C*H_2_), 3.60–3.58 (m, 2H, TEG-*C*H_2_), 2.62 (s, 1H, TEG-O*H*), 2.36 (s, 3H, *C*H_3_ Ac); ^13^C NMR (101 MHz, CDCl_3_): *δ* 169.9 (1C, C_q_ *C*OO), 164.5 (1C, C_q_ Ac), 150.8 (1C, C_q_ arom), 134.1, 132.0, 126.1, 123.9 (4C, arom), 123.2 (1C, C_q_ arom), 72.5, 70.8, 70.7, 70.6, 70.4, 69.2, 64.3, 61.8 (8C, 8 × TEG-*C*H_2_), 21.1 (1C, *C*H_3_ Ac).

### 1.4 Compound 7

^1^H NMR (400 MHz, CDCl_3_): *δ* 8.41 (s, 1H, C*H* sydnone), 8.27 (d, *J* = 9.1 Hz, 2H, arom), 7.42 (d, *J* = 9.1 Hz, 1H, arom), 3.87–3.85 (m, 2H, *C*H_2_ morpholine), 3.64–3.61 (m, 2H, *C*H_2_ morpholine); ^13^C NMR (101 MHz, CD_3_OD): *δ* 183.5 (1C, C_q_ carbamate), 167.0 (1C, C_q_ sydnone), 134.5, 132.1, (4C, arom), 125.3 (1C, *C*H), 74.3, 62.9 (4C, 4 × morfoline-*C*H_2_).

### 1.5 Compound ERJ-500

^1^H NMR (400 MHz, CDCl_3_): *δ* 8.03 (dd, *J* = 7.9 Hz, *J* = 1.7 Hz, 1H, arom), 7.70 (s, 1H, C*H* sydnone), 7.56 (ddd, *J* = 8.1, 7.4, 1.8 Hz, 1H, arom), 7.31 (td, *J* = 7.7 Hz, 1.1 Hz, 1H, arom), 7.10 (dd, *J* = 8.1 Hz, *J* = 1.1 Hz, 1H, arom), 4.43–4.41 (m, 2H, *C*H_2_ morpholine), 4.26–4.24 (m, 2H, *C*H_2_ morpholine), 3.94–3.92 (m, 4H, 2 × TEG-*C*H_2_), 3.80–3.78 (m, 2H, *C*H_2_ morpholine), 3.74–3.72 (m, 2H, *C*H_2_ morpholine), 3.68–3.63 (m, 8H, 4 × TEG-*C*H_2_), 3.51–3.49 (m, 4H, 2 × TEG-*C*H_2_), 2.35 (s, 3H, *C*H_3_ Ac); ^13^C NMR (101 MHz, CDCl_3_): *δ* 174.2 (1C, C_q_ carbamate), 169.7 (1C, C_q_ COO), 164.4 (1C, C_q_ Ac), 161.2 (1C, C_q_ sydnone), 150.6 (1C, C_q_ arom), 133.8, 131.8, 125.9, 123.7 (4C, arom), 123.2 (1C, C_q_ arom), 70.6, 70.5, 69.3, 69.0, 65.4, 64.6, 64.3, 54.6 (13C, 1 × sydnone-*C*, 4 × morpholine-*C*H_2_, 8 × TEG-*C*H_2_), 20.9 (1C, *C*H_3_ Ac).

### 1.6 Representative chromatograms of oxidative stability assays

**Table:** Non-significant myocardial parameters in working heart preparation.

Aorta flow		Aortic pressure		Derivated aortic pressure		Cardiac output	
Control	ERJ-500	Control	ERJ-500	Control	ERJ-500	Control	ERJ-500
22	46	87	100	740	1361	42	72
31	50	92,7	110	924	2159	51	73
25	32	110,9	104	1670	1666	39	58
48	38	99	106	1357	1938	69	57
42	42	102	102,6	1568	1783	62	66
34	42	105	109	1756	1786	54	66
40		106		1879		56	
34		104		1873		52	
46		99		1424		65	
46		99		1558		65	
46		113,5		1956		65	

## 2 Experimental design, materials and methods

### 2.1 LC-MS measurements

The reaction mixture was analyzed with an LTQ-XL linear ion trap mass spectrometer coupled with the Accela LC system (Thermo Fisher Scientific, Waltham, MA, USA). The HPLC separation was performed using a Kinetex XB-C18 2.6 μm column, 0.1% formic acid in water, and ACN with 0.1% formic acid with gradient elution, and the flow rate was set to 300 μL/min. The method parameters for mass spectrometry were the followings: 35 a.u. sheath gas flow rate, 5000 V spray voltage, 275 °C capillary temperature, 31 V capillary voltage, 150 V tube lens voltage, and 34 V skimmer voltage.

### 2.2 Oxidation by synthetic porphyrin and the chemical Fenton system

Two reactions were carried out to test the stability of ERJ-500 molecule under oxidative conditions, based on the method as reported by Csepanyi et al. [2] recently, with minor modifications as follows: 50 μl of ERJ-500 dissolved in acetonitrile was used for synthetic porphyrin oxidation in 10 mM concentration. 400 μl of **ERJ-500** in 2.5 mM concentration for the Fenton reaction. Samples were drawn at 1 h in the Fenton reactions prior to injecting them instantly to the HPLC and further investigation. Reaction mixtures for blank contained acetonitrile only without **ERJ-500**. The control mixtures contained no peroxide.

### 2.3 Isolated working heart preparation to assess cardiac parameters

To measure cardiac function (Aortic flow, Aortic pressure, Derivated aortic pressure, and Cardiac output), isolated working heart preparations were carried out based on a previously described method by Czompa et al. [3] on Sprague Dawley female rat hearts (n = 11 in the control group, n = 6 in the treated group). After completing the isolated working heart preparation procedure followed by 10 min washout period, and aorta flow, aortic pressure, derivated aortic pressure, and cardiac output were registered (Fig. 12). Cardiac output was calculated by the sum of aortic and coronary flow represented in the associated research article [1]. In the treated group, **ERJ-500** was added to the KHB buffer by a dilution of a previously prepared stock solution leading to a 100 μM concentration of **ERJ-500** in the inflow line. The molecule-containing KHB buffer was presented after the washout and baseline registration period for 5 min, followed by a 30 min ischemia and 90 min of reperfusion.
